# Comparison of Modified Cartilage Shield Tympanoplasty with Tympanoplasty Using Temporalis Fascia Only: Retrospective Analysis of 142 Cases

**DOI:** 10.1155/2016/8092328

**Published:** 2016-07-31

**Authors:** Sohil Vadiya, Vibhuti Parikh, Saumya Shah, Parita Pandya, Anuj Kansara

**Affiliations:** Pramukhswami Medical College and Shree Krishna Hospital, Karamsad, Gujarat 388325, India

## Abstract

The current study compares outcomes of modified cartilage shield tympanoplasty (CST) with temporalis fascia tympanoplasty in type I procedures in Indian patients. Graft uptake rates are better with the CST technique and hearing results are almost equivalent with both techniques except at 8000 Hz where improvement in hearing was found better with the use of temporalis fascia alone. The CST technique used in the study is unique.

## 1. Introduction

Cartilage shield technique of tympanoplasty (CST) has shown many promising results so far in various studies [1, 2] and has added a dependable alternative to conventional use of only fascia to obtain indisputable success rates of tympanic membrane reconstruction. The current study aims at comparing graft uptake rates and hearing improvement in temporalis fascia (TF) tympanoplasty and modified CST in Indian patients at a tertiary care center in rural area by analysis of 142 surgeries and their follow-up findings from retrospective data. Yetiser and Hidir [3] have concluded that the hearing gain in patients with cartilage shield grafting was better than that in those who had temporalis fascia tympanoplasty, although experimental analysis shows loss of acoustic energy for thick and large shield cartilage grafts. Fascia grafts are unstable on one hand making graft uptake results variable, whereas with cartilage being thick there is always a concern regarding compromise on hearing gain. This study evaluates retrospective data to give more objectivity to derive conclusions regarding this dilemma and should guide the otologists to employ an appropriate technique with confidence keeping the positive consequences in mind.

## 2. Material and Methods

A total of 142 tympanoplasty surgeries between June 2012 and February 2015 were studied. 65 cases were included in group A where modified cartilage shield tympanoplasty was performed and 77 cases were included in group B where temporalis fascia only was used as the grafting material. Only adult cases with mucosal type of disease with perforation size more than 5 mm in at least one dimension (measured by putting graph paper over the perforation) and where all ossicles were intact and mobile (type I procedure) were included in the study. Preoperative oto endoscopy pictures and pure tone audiogram (PTA) findings were noted and compared with 2 months' and 6 months' postoperative PTA and oto endoscopy findings. All surgeries were performed by the same surgeon (first author) and the same technique was used in all cases. The technique used in this study for modified cartilage shield is unique and different from many other techniques.

**Figure 1:**
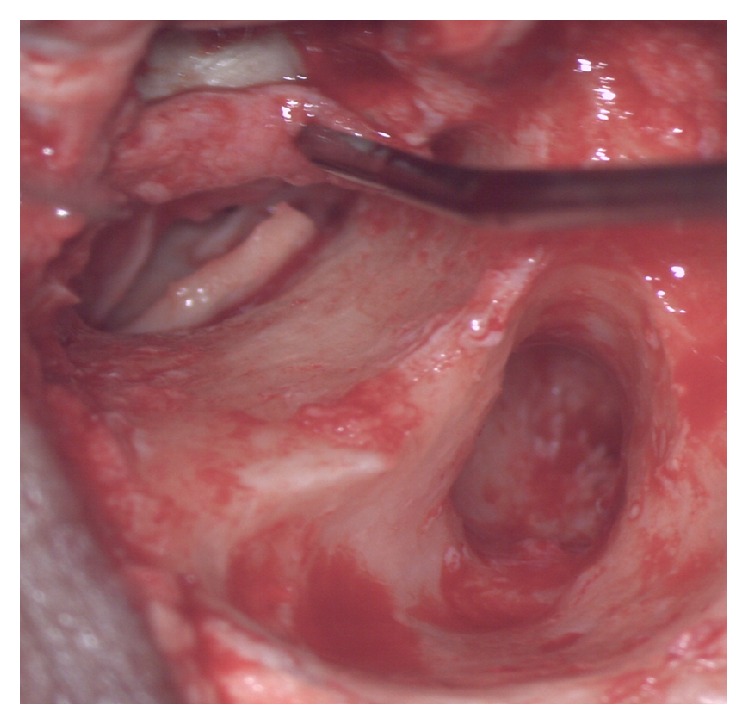
Cartilage piece being kept medial to the handle of malleus.

For all the cases in both groups, postauricular incision was used. Vascular strip incision with anterior tucking (VSAT) technique of canal wall incision [4] was used in all cases in both groups. Temporalis fascia graft was harvested and, after canal wall incisions, middle ear was explored after lifting the annulus and intactness of all ossicles was confirmed. Cases with ossicular erosion were excluded from the study. Cortical mastoidectomy was done in all the cases of both groups and aditus patency was achieved. For group A patients, tragal cartilage was harvested and perichondrium kept attached on one side (to be placed laterally) and removed from the other side. Cartilage was made thin to almost half thickness with the help of number 15 surgical blade and knife. Notch was never made in the cartilage piece. This will make the final thickness of the cartilage about 0.5 mm. Cartilage piece was kept lateral to the incudostapedial joint and medial to the handle of malleus (Figure 1). TF was placed over the cartilage and also lateral to the handle of malleus but medial to the annulus. Anterior tucking of the fascia was done in all cases. Abundant gelfoam was kept inside middle ear to support the cartilage piece and also laterally in the external auditory canal. In group B cases, temporalis fascia was kept lateral to the handle of malleus and medial to the annulus as an over-under grafting, as recommended by Kartush et al. [5]. Anterior tucking of fascia was also done. Cartilage was not used in these group B cases. It is important that the handle of malleus is separated from remnants of tympanic membrane (TM) in order to make it easy and safe to put fascia lateral to the handle but medial to the remnants of TM and to the annulus. Abundant gelfoam was kept medial and lateral to the grafted fascia. All cases where regular follow-up was maintained were studied. Preoperative results compared with postoperative results at 6 months' follow-up.

## 3. Results

**Figure 2:**
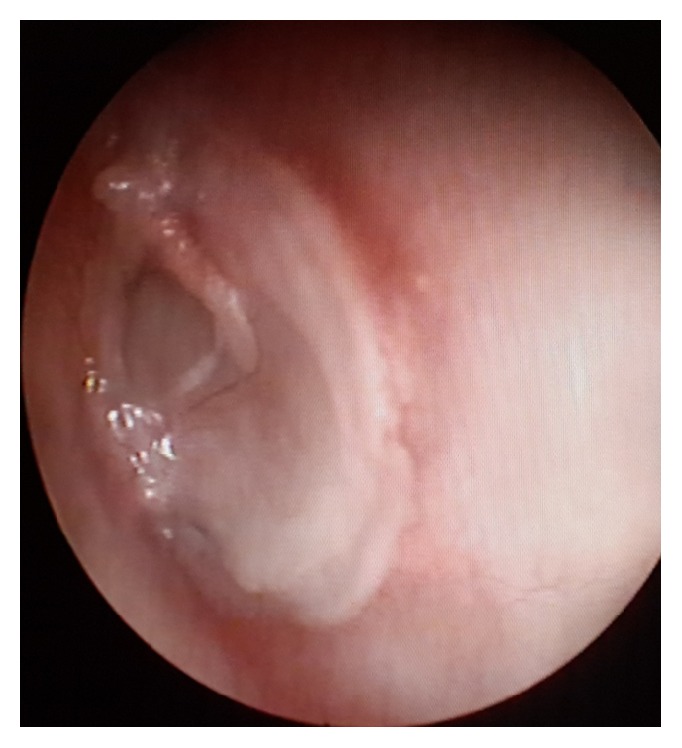
Postoperative picture at 6 months after modified cartilage shield technique.

**Figure 3:**
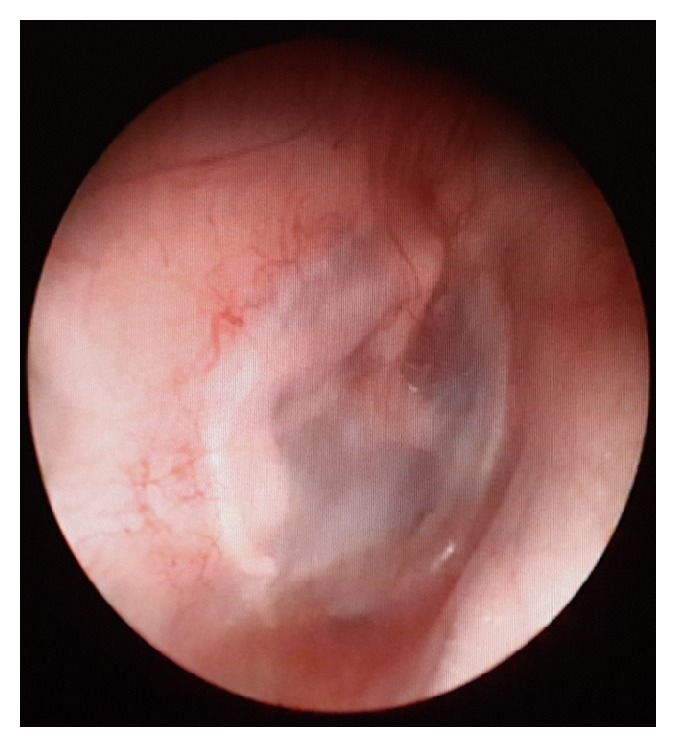
Postoperative picture at 6 months after temporalis fascia tympanoplasty.

63 patients and total 65 ears were operated on with the modified CST technique and these were included in group A. 77 cases were operated on with TF method of tympanoplasty and were included in group B. All patients were between 20 and 50 years of age. Patients with diabetes were not included in the study. 64 out of 65 cases in group A and 69 out of 77 cases in group B showed successful graft uptake (Figures 2 and 3) (Table 1).

One patient in group A had residual perforation at 2 months' follow-up. So graft uptake rate for group A is 98.46%. Seven cases in group B required a revision procedure and one case had small residual perforation that healed with conservative means, so graft uptake for group B is 89.61%. Statistical analysis of graft uptake rates shows *P* value of 0.031025 with the help of chi-square test, suggestive of statistically significant better graft uptake rates in group A cases as compared to group B cases.

Improvement in hearing at various frequencies in both groups is depicted in Table 2.

It is very much evident that hearing improvement in both groups is almost similar except at 8000 Hz frequency where improvement in group B is better than group A. At other frequencies, the difference is statistically not significant between the two groups.

Student's *t*-test with unequal variances was used for statistical analysis. Average pre-op ABG at speech frequencies (mean of 500, 1000, and 2000 Hz) in group A was 33.99 db and in group B it was 34.48 db and average post-op ABG was found to be 16.23 db in group A and 17.05 db in group B with 60 cases (92.30%) in group A and 74 (96.10%) cases in group B having less than 20 db ABG at 6 months post-op. Average pre-op ABG in group A at 8000 Hz was 32.69 ± 7.0 db and post-op average ABG was 22.46 ± 7.0 db, whereas in group B, average pre-op ABG at 8000 Hz was 32.33 ± 7.0 db and post-op average ABG of 17.66 ± 7.0 was noted. No lateralisation or medialisation of graft was seen in any cases in any groups in this study. Deterioration of bone conduction indicative of sensorineural hearing loss was not seen in any of the cases in both groups. Possibility of compromise regarding less hearing gain at high frequency with modified cartilage shield technique should be explained to the patients before surgery and an informed consent should be taken for the same before surgery. No other major complications were noted in any groups.

## 4. Discussion

This study was designed to evaluate results of tympanoplasty and to see if there is significant difference in hearing when cartilage shield tympanoplasty is used for reconstruction of tympanic membrane and also to note the graft uptake rates. Many patients of chronic otitis media in India have unhealthy middle ear mucosa and large, subtotal, or total perforations of the ear drum. This requires additional support for the graft material to increase chances of graft uptake. The current study was done in cases of mucosal disease to objectively evaluate results of tympanoplasty and to study if there is any difference in hearing improvement as cartilage is a thicker grafting material.

Aarnisalo et al. [11] have concluded that the placement of cartilage on the medial surface of TM reduces the motion of the TM that opposes the cartilage. These obvious local changes occur even though the cartilage had little effect on the sound-induced motion of the stapes. Mohamad et al. [12] have concluded that tympanoplasty using cartilage with or without perichondrium has better morphological outcome than tympanoplasty using temporalis fascia. However, there was no statistically significant difference in hearing outcomes between the 2 grafts. Khan and Parab [13] have shown good anatomical and functional results using sliced cartilage for tympanoplasty technique. Chhapola and Matta [14] have mentioned that cartilage thickness of <0.5 mm is seen to have similar acoustic properties as the tympanic membrane. The current study shows better graft uptake rates with the use of cartilage shield method and hearing improvement comparable with TF as graft material (Table 3). The technique used in this study for modified cartilage shield is unique.

## 5. Conclusion

Modified cartilage shield tympanoplasty offers better graft uptake rates as compared to temporalis fascia alone. Hearing improvement in modified cartilage shield tympanoplasty is almost equivalent to temporalis fascia tympanoplasty except at 8000 Hz, where hearing improvement in fascia grafted ears is better with statistically significant difference. However, before surgery the possibility of compromise regarding less hearing gain at high frequency with modified cartilage shield technique should be explained to the patients and an informed consent should be taken for the same before surgery.

## Figures and Tables

**Table 1:** Graft uptake rates of patients in both groups.

	Group A	Group B	Total
Successful	64	69	133
Unsuccessful	1	8	9

Total	65	77	142

**Table 2:** Comparison of hearing improvement in both groups.

Frequency (Hz)	Group A average hearing gain (ABG) db (*n* = 65)	Group B average hearing gain (ABG) db (*n* = 77)	*P* value
250	15.0	14.93	0.916, not significant
Mean of 500, 1000, 2000	17.76	17.42	0.585, not significant
4000	15.92	16.75	0.341, not significant
8000	10.23	14.27	<0.0001, significant

**Table 3:** Comparison of success rates of different graft materials in tympanoplasty.

Author	Graft material	Take-up (%)
Dornhoffer [6]	Perichondrium	85
Borkowski et al. [7]	Cartilage, perichondrium	100
Neumann et al. [8]	Cartilage palisade	100
Indorewala [9]	Fascia lata	95
Indorewala [9]	Temporalis fascia (TF)	66
Mundra et al. [10]	TF/perichondrium with cartilage slice	98.94
Present series	Cartilage shield with perichondrium	98.46
Present series	Temporalis fascia (TF)	89.61

## Additional Points

Animals were not involved in this study. This is a retrospective data analysis study.
